# Improved PPO Optimization for Robotic Arm Grasping Trajectory Planning and Real-Robot Migration

**DOI:** 10.3390/s25175253

**Published:** 2025-08-23

**Authors:** Chunlei Li, Zhe Liu, Liang Li, Zeyu Ji, Chenbo Li, Jiaxing Liang, Yafeng Li

**Affiliations:** 1 Shaanxi Key Laboratory of Advanced Manufacturing and Evaluation of Robot Key Components, Baoji 721016, China; 602lcl-602lcl@163.com; 2 School of Mechanical Engineering, Baoji University of Arts and Sciences, Baoji 721016, China; liuzhe19991006@163.com (Z.L.);; 3 School of Computer Science and Technology, Baoji University of Arts and Sciences, Baoji 721013, China

**Keywords:** robotic trajectory planning, proximal policy optimization, simulated annealing, unstructured environments, collision-free grasping, reinforcement learning, sim-to-real transfer

## Abstract

Addressing key challenges in unstructured environments, including local optimum traps, limited real-time interaction, and convergence difficulties, this research pioneers a hybrid reinforcement learning approach that combines simulated annealing (SA) with proximal policy optimization (PPO) for robotic arm trajectory planning. The framework enables the accurate, collision-free grasping of randomly appearing objects in dynamic obstacles through three key innovations: a probabilistically enhanced simulation environment with a 20% obstacle generation rate; an optimized state-action space featuring 12-dimensional environment coding and 6-DoF joint control; and an SA-PPO algorithm that dynamically adjusts the learning rate to balance exploration and convergence. Experimental results show a 6.52% increase in success rate (98% vs. 92%) and a 7.14% reduction in steps per set compared to the baseline PPO. A real deployment on the AUBO-i5 robotic arm enables real machine grasping, validating a robust transfer from simulation to reality. This work establishes a new paradigm for adaptive robot manipulation in industrial scenarios requiring a real-time response to environmental uncertainty.

## 1. Introduction

With the continuous advancement of robotic arm technology, industrial robotic arms have become indispensable assets in modern industrial production. By deploying robotic arms to replace human labor in performing various simple yet repetitive mechanical tasks, such as grasping and placement operations, current productivity levels can be significantly enhanced. In future smart factories, work environments will exhibit dynamic and unstructured characteristics, requiring robotic arms to operate efficiently and safely while performing complex tasks [1]. However, with the growing demands in industrial production and service sectors, existing robotic grasping technologies can no longer meet future requirements. There is an increasing expectation for robotic arms to possess autonomous motion capabilities to handle various environmental uncertainties [2]. Consequently, research on reinforcement learning-based trajectory planning for robotic arms carries significant importance.

Robotic grasping tasks exhibit remarkable versatility in industrial applications, with distinct operational scenarios dictated by varying environments and target objects. These systems also play pivotal roles in human–robot interaction and service robotics domains [3,4]. Diverse trajectory planning methods have been developed for robotic arms across different application scenarios. Conventional kinematic and dynamic planning approaches [5], which generate trajectories through mathematical analysis or numerical computation based on the arm’s kinematic model, prove effective for deterministic tasks in structured environments. However, their heavy dependence on precise kinematic modeling results in poor adaptability to unstructured environments and limited capability in handling dynamic obstacles. Geometry-based path planning algorithms [6] model the robotic arm’s workspace as geometric constructs, generating collision-free paths through graph search or sampling methods. While effective in principle, these approaches suffer from high computational complexity and often fail to guarantee globally optimal solutions. In contrast, intelligent optimization algorithms [7] employ bionic principles or mathematical optimization to search for optimal trajectories without relying on precise models, making them suitable for complex constrained optimization problems. However, these methods exhibit notable limitations, including slow convergence rates, susceptibility to local optima, and the need for manual parameter tuning. While these methods demonstrate application value across various scenarios, they inherently possess limitations. Recent advances in machine vision have endowed robotic arms with environmental perception capabilities, significantly enhancing their intelligence. Consequently, the development of grasping systems capable of handling unknown objects in unconstrained environments has emerged as a key research focus.

To overcome the limitations of existing approaches while leveraging their respective strengths, reinforcement learning [8] offers a promising solution for robotic grasping. Through continuous agent–environment interactions, this approach eliminates the need for manual annotation by utilizing predefined reward signals as feedback mechanisms, thereby enabling autonomous environmental perception. Reinforcement learning has demonstrated significant practical achievements across diverse domains, including autonomous vehicle control [9], cross-lingual translation, speech recognition, biomimetic agents, game playing (Go and esports), and robotic arm manipulation. These advancements have created new opportunities for integrating virtual reality and digital twin technologies into intelligent control systems [10,11], thereby enabling novel approaches to robotic arm intelligent control. In 2013, Mnih et al. [12] integrated Q-learning with convolutional neural networks, introducing the groundbreaking Deep Q-Network (DQN) framework. The team subsequently demonstrated the efficacy of this approach through successful applications in gaming environments [13]. Wang et al. [14] modified the DQN architecture by splitting it into two separate pathways for agent action selection and Q-value generation respectively. In an alternative approach, Hasselt et al. [15]. improved DQN through a dual-network structure. The research group led by Li Heyu [16] proposed a robotic arm control method based on the deep deterministic policy gradient algorithm, creating a Unity3D simulation environment that successfully demonstrated object grasping in production line scenarios. Researchers at UC Berkeley led by Schulman [17] proposed the Trust Region Policy Optimization (TRPO) algorithm, whose core innovation involves modifying the cost function to enable better policy generation. The same team later developed the Proximal Policy Optimization (PPO) algorithm [18], the experimental results of which showed it could enhance training stability without compromising efficiency.

To achieve successful robotic grasping and efficient training, this study establishes a 1:1 simulated environment using PyBullet, incorporating models of the robotic arm, gripper, gantry system, and cameras. Building upon PPO, we optimize the state space, action space, and reward mechanism. The training process integrates simulated annealing (SA) to dynamically adjust learning rates, initially assigning higher values to enhance exploration and escape local optima during early training phases. The learning rate is dynamically adjusted with the increase in the number of training rounds during training, and converges stably in the middle and late stages to achieve efficient learning and be able to train a model that can accomplish the grasping task. After obtaining the model, sim-to-real experiments were conducted. By addressing issues such as coordinate transformation, normalization, and data acquisition between the real and simulated environments, the model was successfully transferred to a real robotic arm.

The results of this experiment demonstrate the ability to achieve autonomous target recognition, grasping, and obstacle avoidance in a simulated environment, aiming to address the aforementioned shortcomings and enabling application in material handling operations performed by robotic arms in factories. We aim to enable robotic arms to automatically identify and grasp target materials in the unstructured or semi-structured environments commonly found in factory workshops. Ultimately, this research seeks to develop more adaptive, safer, and easier-to-deploy robotic grasping solutions for manufacturing, reducing reliance on fixed automated systems and lowering the implementation barriers for robotic automation in complex material handling scenarios. In the field of embodied intelligence, the results of this experiment can also provide a motion model foundation for tasks.

## 2. Related Work

Although the PPO algorithm improves stability by adjusting the objective function, its monotonous policy update mechanism is prone to fall into local optimality in high-dimensional continuous control tasks. Xing Chen et al. [19] demonstrated through a non-strategy metric that the success rate of the PPO algorithm in reward-sparse scenarios (e.g., multi-obstacle grasping) is less than 40%, and there is a limited space of strategies that can be explored. In addition, the existing PPO improvement schemes that integrate Simulated Annealing (SA) have the problem of decoupling between parameters and the environment. Taking the COAPG-PPO algorithm proposed by Zhou Yi et al. [20] as an example, the fixed annealing scheme adopted by this algorithm reflects this problem. It leads to insufficient exploration in the early stage of the algorithm’s operation, and excessive disturbance in the later stage, which in turn triggers policy oscillations. In meeting the growing demand for more flexible and intelligent automation technologies in modern manufacturing, especially in the field of material handling tasks, current solutions typically rely on pre-programmed trajectories or specialized fixtures that are inflexible, require significant engineering resources to adapt to new tasks or components, and struggle to cope with dynamic environments or unexpected obstacles. We identified the main limitations of existing deployments, as follow: (1) it is challenging to reliably grasp random objects without extensive re-engineering; (2) the lack of intrinsic obstacle avoidance poses a safety risk and operational inefficiencies when human workers or other equipment are present; and (3) the high cost and time required to program and debug a new task hampers adaptability to small-quantity, multi-variety production.

To address the above deficiencies, this study proposes an adaptive PPO + SA framework with an innovative mechanism, which optimizes the reward and punishment mechanism of PPO, adjusts the parameters to achieve the function of obstacle-avoidance grasping and establish an exponential correlation between the temperature and the strategy entropy, realizes the self-attenuation of the exploration intensity with the convergence of the strategy, which makes it easier to jump out of the local optimum at the initial stage, and finally migrates the model obtained from training to a real machine for random target grasping or obstacle avoidance grasping, which fundamentally solves the local convergence problem of PPO and non-adaptive SA.

## 3. Optimized PPO Algorithm Architecture for Robotic Arm Grasping

Advances in machine vision have endowed robotic arms with environmental perception capabilities. Reinforcement learning further enhances this perception through continuous environmental interaction. This study optimizes the state space, action space, reward function, and penalty function in the PPO algorithm. The trained model enables collision-free random grasping by robotic arms, establishing a foundation for real-world grasping applications.

### 3.1. Proximal Policy Optimization (PPO)

**Figure 1:**
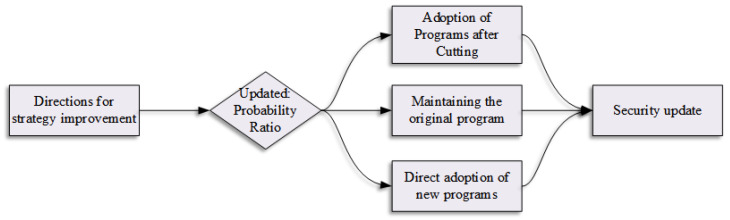
Illustration of the probability ratio trimming mechanism of the PPO algorithm.

As a simplified variant of the TRPO algorithm, PPO incorporates a probability ratio clipping mechanism. This approach maintains training stability while significantly reducing computational overhead, establishing PPO as both efficient and easily implementable. Figure 1 illustrates the schematic of this probability ratio clipping mechanism.

In PPO, the policy represents a probability distribution over the agent’s actions, while the value function evaluates the current policy’s performance. The complete objective function is given by Equation (1):

(1) $$L\left(\theta \right)=E_{t}\left[L^{CLIP}\left(\theta \right)-c_{1}\times VF\left(\theta \right)+c_{2}\times S\left[\pi \theta \right]\right]$$

In the above equation, $L^{CLIP}\left(\theta \right)$ denotes the clipped policy objective, which constrains policy updates by clipping the probability ratio to prevent excessive deviation from the previous policy, thereby maintaining training stability. VF(θ) represents the value function loss term (typically implemented as mean squared error) for training the state-value baseline. S[πθ] is the policy entropy term that encourages exploration. The coefficients $c_{1}$ and $c_{2}$ balance the relative contributions of these loss components.

### 3.2. State and Action Space Design

The state space characterizes the current environmental features and serves as input to the policy network. For the six-degree-of-freedom robotic arm grasping task, the designed state vector s integrates critical features encompassing the arm’s kinematic state, target object information and environmental constraints to form a 12-dimensional representation, and the observed state space is shown in Equation (2):

(2) $$s={\left[q_{1},q_{2},q_{3},q_{4},q_{5},q_{6},x_{g},y_{g},z_{g},x_{t},y_{t},z_{t},x_{o},y_{o},z_{o},\Delta p_{o},\theta_{gz}\right]}^{T}$$

The observed state space integrates six core components essential for robotic grasping. The joint angles $\left[q_{1},q_{2},q_{3},q_{4},q_{5},q_{6}\right]$ represent current angular positions of all six rotational joints to fully capture the arm’s kinematic configuration, while the end-effector coordinates $\left[x_{g},y_{g},z_{g}\right]$ specify its spatial position through the gripper center’s 3D global coordinates. Crucially, the target coordinates $\left[x_{t},y_{t},z_{t}\right]$ define the desired grasping location, complemented by obstacle coordinates $\left[x_{o},y_{o},z_{o}\right]$ mapping environmental constraints. The proximity metric $\left[\Delta p_{o}\right]$ encodes the minimum joint-to-obstacle distance as a critical safety parameter, and the orientation angle $\left[\theta_{gz}\right]$ between the end-effector and world z-axis provides essential posture feedback. Collectively, these elements enable the policy network to synthesize real-time joint kinematics, precise tool positioning, target localization, and environmental awareness into actionable control decisions.

**Figure 2:**
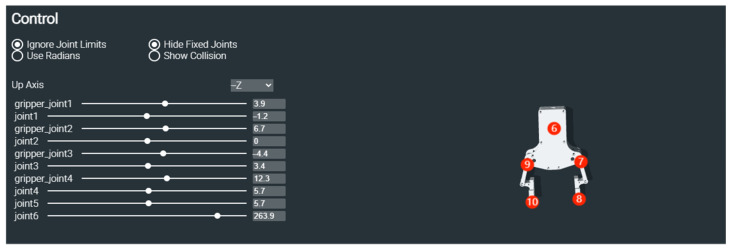
Representation of jaw linking points.

The gripper’s center point is determined through analysis of the linkage configuration shown in Figure 2, where circled numbers indicate joint indices defined in the Unified Robot Description Format (URDF) file [21]. The URDF provides standardized kinematic and dynamic modeling for robotic systems. This study defines the gripper’s operational center as the midpoint between Link 7 and Link 10, selected for their symmetrical positioning and mechanical dominance in grasping operations.

**Figure 3:**
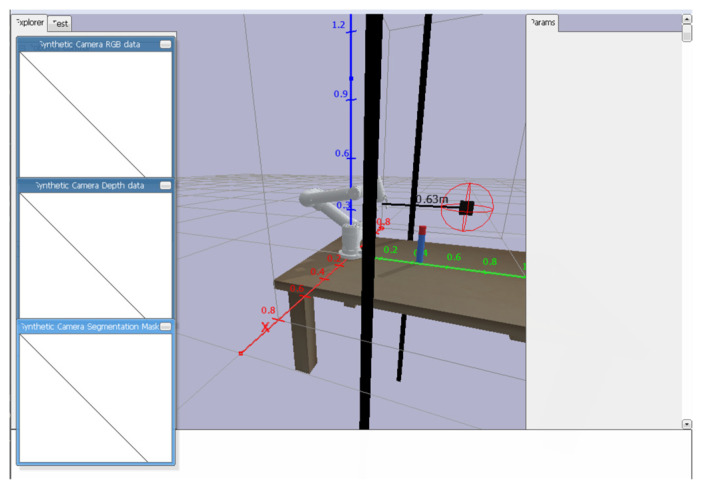
Minimum distance of clamping jaws from random obstacles and radiation map of random obstacles.

The obstacle-aware system combines the 3D coordinates of an obstacle with the minimum distance between each robot joint and the obstacle, allowing for more efficient obstacle avoidance learning. A collision will immediately trigger the maximum penalty and terminate the current round of training that caused the collision, proceeding directly to the next round, while near misses (e.g., with a gap of less than 5 cm) are penalized proportional to the distance without interrupting training—the closer the distance, the heavier the penalty. This hierarchical reward structure strictly enforces safety constraints while preserving valuable training episodes, thus promoting a robust obstacle avoidance grasping strategy. The dual spatial representation (absolute coordinates + relative distances) provides comprehensive environment awareness, and the graded punishment scheme provides a clear optimization gradient for the strategy network. As illustrated in Figure 3, obstacle avoidance for the gripper is demonstrated. The spherical volumes outlined in red represent the influence zones of randomly generated obstacles, while the black line indicates the shortest distance between the robotic gripper and the nearest obstacle. Since the current minimum distance is measured relative to the obstacle marked by the red sphere, other randomly placed obstacles are disregarded in the immediate computation. During arm movement, this minimum distance is continuously recalibrated in real time, enabling comprehensive collision-free grasping through dynamic obstacle awareness. The system’s focus on the most critical obstacle (nearest violation risk) ensures computational efficiency while maintaining safety.

**Figure 4:**
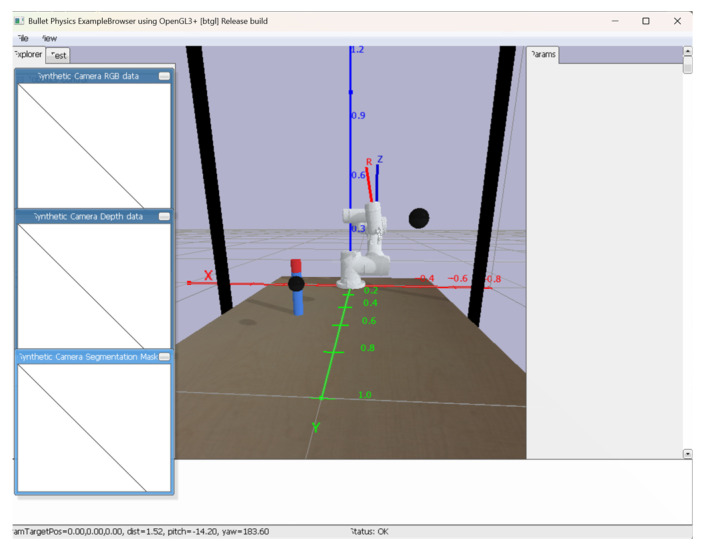
Schematic diagram of the angle between the end of the jaws and the *z*-axis of the world coordinate system.

The system regulates vertical grasping through environmental constraints by calculating the angle between the end-effector and the global *z*-axis. This angular deviation serves as the primary metric for vertical alignment assessment, where smaller angles indicate closer conformity to ideal perpendicular grasping conditions. The implementation continuously monitors this orientation during approach phases, enabling real-time trajectory adjustments to achieve optimal perpendicular contact with target objects. As shown in Figure 4, the red line “R” indicates the orientation of the robotic arm’s end-effector, while the blue line “Z” represents the *z*-axis, which is consistent with the *Z*-axis of the world coordinate system and is used to observe the offset angle more intuitively. The angle between the red line and blue line is calculated, and when this angle exceeds a certain threshold, fine-tuning intervention will be performed. The adjustment continues until the angle falls below the predefined threshold, which confirms that the gripper’s end-effector has reached an approximately vertical state.

A six-degree-of-freedom (6-DoF) robotic arm is the standard kinematic configuration for industrial manipulation tasks, capable of positioning the end-effector at any three-dimensional spatial coordinate and achieving the desired posture. This capability stems from its articulated structure composed of six rotary joints—typically arranged in a serial mechanism configuration—enabling the independent control of Cartesian positions (x, y, z) and attitudes (roll, pitch, yaw). The industrial models used in this study, such as the AUBO-i5, feature a spherical workspace with a radius of approximately 886.5 mm and a repeatability accuracy of ±0.02 mm, making them an ideal choice for precise operations in unstructured environments. The action space describes the behavior output by the policy network and serves as the control command for the robotic arm. For the continuous motion control task of a 6-DoF robotic arm, this paper selects joint angle variations as the action output, constructing a 6-dimensional continuous action space a, as shown in Equation (3):

(3) $$a={\left[∆q_{1},∆q_{2},∆q_{3},∆q_{4},∆q_{5},∆q_{6}\right]}^{T}$$

where $\left[∆q_{1},∆q_{2},∆q_{3},∆q_{4},∆q_{5},∆q_{6}\right]$ represent the angular increments of the six rotary joints, controlling the robotic arm’s joint movements at each step. Additionally, the incremental value $∆q_{i}$ for each joint angle is constrained within a specified range to ensure the physical feasibility of the actions,

(4) $$∆q_{i}\in \left[-∆q_{max},∆q_{max}\right]$$

In Equation (4), $∆q_{max}$ denotes the maximum allowable angular increment per step for each joint. To integrate state and action, at each moment t, the robotic arm generates action $a_{t}$ through the policy network based on the current state $s_{t}$, as expressed in Equation (5).

(5) $$a_{t}=\pi_{\theta }\left(s_{t}\right)$$

In this equation, $\pi_{\theta }\;$ represents the policy network, and its parameters θ are updated using an improved algorithm that combines PPO with simulated annealing. Finally, after receiving the action $a_{t}$, the robotic arm updates its joint angles, resulting in a new state $s_{t+1}$, while the environment provides corresponding reward feedback.

The 12-dimensional state vector comprehensively incorporates the robotic arm’s joint angles, end-effector pose, target position, and environmental obstacle information. This design ensures the policy network can fully capture task-critical features, thereby enabling the efficient learning of complex grasping maneuvers.

### 3.3. Loss Function and Reward Design

In the optimized PPO algorithm, the design of the loss function and reward function is critical for enhancing the performance of the 6-DoF robotic arm in grasping tasks. A well-structured loss function ensures stable policy network updates, while an effective reward function guides the robotic arm toward optimal action strategies. The PPO algorithm’s core objective function, serving as the foundational loss function, is defined in Equation (6).

(6) $$L_{PPO}\left(\theta \right)=E_{t}\left[\min\left(r_{t}\left(\theta \right)A_{t},clip\left(r_{t}\left(\theta \right),1-\in ,1+\in \right)A_{t}\right)\right]$$

In the above equation, $r_{t}\left(\theta \right)$ denotes the probability ratio of the old and new strategies, $A_{t}$ is the dominance function, which measures the advantage of the current action over the baseline strategy, and ∈ is the trimming threshold, which is used to limit the magnitude of the strategy update to prevent drastic fluctuations in the training process.

To guide the 6-DoF robotic arm in efficiently performing grasping tasks, we design a compound reward mechanism. A significant positive reward is provided only upon successful grasping of the target object, creating a sparse but clear success signal. Collisions between the robotic arm and the environment or target object trigger a strong negative penalty to prevent harmful motions. Additionally, a distance-based reward incrementally encourages the end-effector to approach the target, reducing inefficient exploration, while an obstacle-avoidance reward ensures safe operation by penalizing proximity to obstacles. This multi-faceted reward structure balances task completion, motion safety, and operational efficiency.

The gripper distance reward can be defined by Equation (7), where d represents the distance between the gripper and the target, while ∆d denotes the reduction in this distance, and $d_{grab}$ represents the maximum distance at which the robot arm gripper can successfully grasp the target object. Within this distance range, tests have shown that the target object can be successfully grasped. The closer the distance, the better the grasping effect. Therefore, even if this distance has been reached, we still encourage further approach. A larger ∆d indicates that the gripper is progressively approaching the target. ratio serves as a distance coefficient, and RC is a reward constant introduced to address the diminishing incentive issue. When the distance becomes sufficiently small, even with a large ratio coefficient, the product ratio×∆d would yield an insignificant value due to the minimal ∆d, which fails to adequately encourage further approach. To overcome this limitation, the reward constant RC is incorporated to provide sustained motivation when the gripper is in close proximity to the target.

(7) $$R_{distance}=\left\{\begin{array}{c} RC+ratio\times ∆d,\;d\leq d_{grab}\\ ratio\times ∆d\;,\;d>d_{grab} \end{array}\right.$$

The formula for the successful gripping reward of the clamping jaws is shown in Equation (8).

(8) $$R_{success}=\left\{\begin{array}{c} +1,The\;gripping\;jaws\;grasp\;the\;target\;object\\ -1,Robotic\;arm\;collision\;or\;too\;long\;execution\;steps \end{array}\right.$$

The gripper jaw grasping obstacle avoidance reward formula is shown in Equation (9).

(9) $$R_{obstacle}=\left\{\begin{array}{c} +1,Robotic\;arm\;approaching\;the\;target\;from\;a\;safe\;distance\;away\\ -1,\mathrm{Robotic}\;\mathrm{arms}\;\mathrm{collide}\;\mathrm{with}\;\mathrm{obstacles}\;\mathrm{or}\;\mathrm{do}\;\mathrm{not}\;\mathrm{meet}\;\mathrm{safety}\;\mathrm{distances} \end{array}\right.$$

The final composite reward function integrates these components through Equation (10), where $F_{distance}$, $F_{success}$ and $F_{obstacle}$ denote the factor of the distance reward, success reward, and obstacle avoidance reward, respectively. These factors are dynamically adjusted: $F_{distance}$ is progressively reduced from 2.0 to 1.0, $F_{success}$ is progressively increased from 0.5 to 1.5, and $F_{obstacle}$ is fixed at 0.5. In early training when the robotic arm has not yet mastered the grasping skill, this dynamically adjusted coefficient scheme prioritizes shorter distances to minimize the effect of sparse success/failure signals. As training advances and the end-effector approaches the target, the increasing emphasis on success rewards promotes a grasping behavior that completely avoids obstacles. Most importantly, the reward for obstacle avoidance is smaller than the distance reward to prevent the robotic arm from over-prioritizing obstacle avoidance at the expense of approaching the target.

(10) $$R=F_{distance}\times R_{distance}+{F_{success}\times R}_{success}+F_{obstacle}\times R_{obstacle}$$

The proposed design offers significant advantages through its multidimensional state space, which comprehensively incorporates both the robotic arm’s intrinsic state and extrinsic environmental information (target objects and obstacles). This serves as the critical foundation for enabling collision-free random grasping. The action space is strategically decomposed; joint0, joint1, and joint2 primarily drive the arm toward the target, while joint3 and joint4 execute fine adjustments for vertical grasping alignment, and joint5 rotates the gripper to the optimal grasping angle. This hierarchical actuation ensures efficient and precise target acquisition. Furthermore, the optimized reward—penalty mechanism systematically guides the arm to learn essential skills—including target approach, vertical grasping, and obstacle avoidance—throughout the training process. This results in a robotic system that simultaneously achieves safety (collision prevention), global robustness (adaptation to varying initial conditions), and task adaptability (generalization to unseen scenarios). The synergistic integration of these components guarantees reliable performance in complex grasping tasks.

## 4. Improved PPO Algorithm Design Based on Simulated Annealing Algorithm

The optimized PPO algorithm described above has yielded a preliminary trajectory planning model for grasping tasks capable of reproducing basic operations. However, several limitations persist, including insufficient initial exploration, susceptibility to local optima, and convergence difficulties. To address these issues, this study proposes an improved PPO algorithm incorporating simulated annealing principles. Simulation experiments demonstrate measurable improvements in overcoming these challenges.

### 4.1. Simulated Annealing (SA)

The simulated annealing algorithm [22] is a widely-used stochastic heuristic optimization method inspired by the thermodynamic annealing process, where a material is gradually a cooled from high temperature to allow its particles to reach the minimum energy state, ultimately forming a crystalline structure. The algorithm introduces a “probability of accepting inferior solutions,” enabling it to escape local optima and eventually converge to a global or near-optimal solution. The probability of accepting a new solution in simulated annealing is given by Equation (11), where ∆E represents the energy difference between the current and new solutions.

(11) $$P=exp(-\frac{∆E}{T})$$

### 4.2. Design of Improved PPO Algorithm for SA Based on Crawling

The SA algorithm aims at global optimization through dynamic parameter tuning, while the PPO algorithm maximizes cumulative rewards through strategy gradients. The basic goal of both is to optimize the decision-making strategy, reflecting theoretical compatibility and complementary exploration—development balance. As mentioned earlier, while PPO exhibits stable performance in complex tasks, its policy update relies on local gradient optimization and thus is prone to local optima in high-dimensional spaces, especially in scenarios involving sparse rewards or multi-objective functions. To address this limitation, this study employs a simulated annealing technique with a dynamically adjustable learning rate. During the initial search phase, this hybrid approach may accept sub-solutions, thereby escaping from local optima while significantly enhancing global search capabilities. The temperature scheduling mechanism in SA creates a natural synergy with the shear objective function of the PPO, creating a balanced optimization process that maintains the training stability of the PPO while overcoming its inherent tendency towards local convergence. This integration proves to be particularly effective for robot manipulation tasks that require both the precise improvement of the strategy and in-depth exploration of the state space. The core strength of the PPO combined with the SA algorithm lies in its controllable, adaptive exploration mechanism (realized through temperature parameters) and its ability to systematically accept temporarily inferior solutions to cross energy barriers.

In the PPO algorithm, SA is primarily employed to dynamically adjust the learning rate, maintaining a higher rate during initial training phases for enhanced exploration while gradually reducing it to facilitate convergence. This study implements an adaptive annealing scheme, where the learning rate adjustment follows Equation (12).

(12) $${lr}_{n}={lr}_{0}\times rf$$

In this formulation, ${lr}_{n}$ denotes the learning rate at the nth update cycle, ${lr}_{0}$ represents the initial learning rate at training commencement, and rf serves as the reduction factor for learning rate decay. The system automatically decreases the learning rate when the monitored performance metric fails to improve over consecutive training cycles, as governed by the above equation. This adaptive mechanism ensures comprehensive training across different learning rate regimes while maintaining optimization stability. The initially high learning rate corresponds to the “high temperature phase” of the SA, which allows for a large parameter update step size, enabling the model to be tuned significantly within the solution space. This aggressive updating strategy facilitates the escape from the initial local optimum and promotes the rapid exploration of different regions. As training proceeds, the system enters the “low-temperature phase” of the SA, characterized by a reduced learning rate, where smaller and more precise parameter adjustments enable fine-tuned convergence.

**Figure 5:**
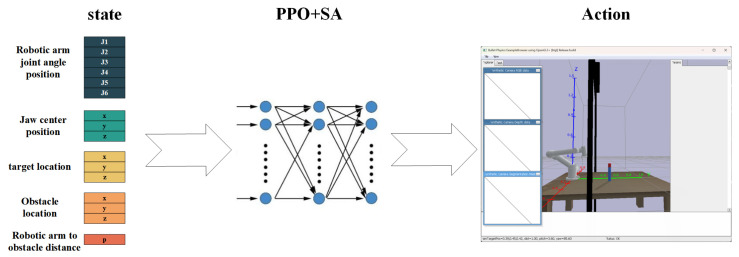
Block diagram of robotic arm trajectory planning training based on PPO + SA algorithm.

The simulation experiment for robotic arm trajectory planning using the SA-enhanced PPO algorithm comprises four key steps, as illustrated in Figure 5. First, a virtual environment capable of real-time training visualization is constructed to demonstrate the solution set. Subsequently, to accomplish the designated tasks, an algorithmic implementation environment is established specifically for model training. During the process, joint angle data are collected as input values, while comparative PPO training is conducted according to the designed reward function, with dynamic learning rate adjustments performed based on training outcomes. Finally, the trained robotic arm joint parameters are saved and deployed in the simulation environment to execute the target tasks.

**Figure 6:**
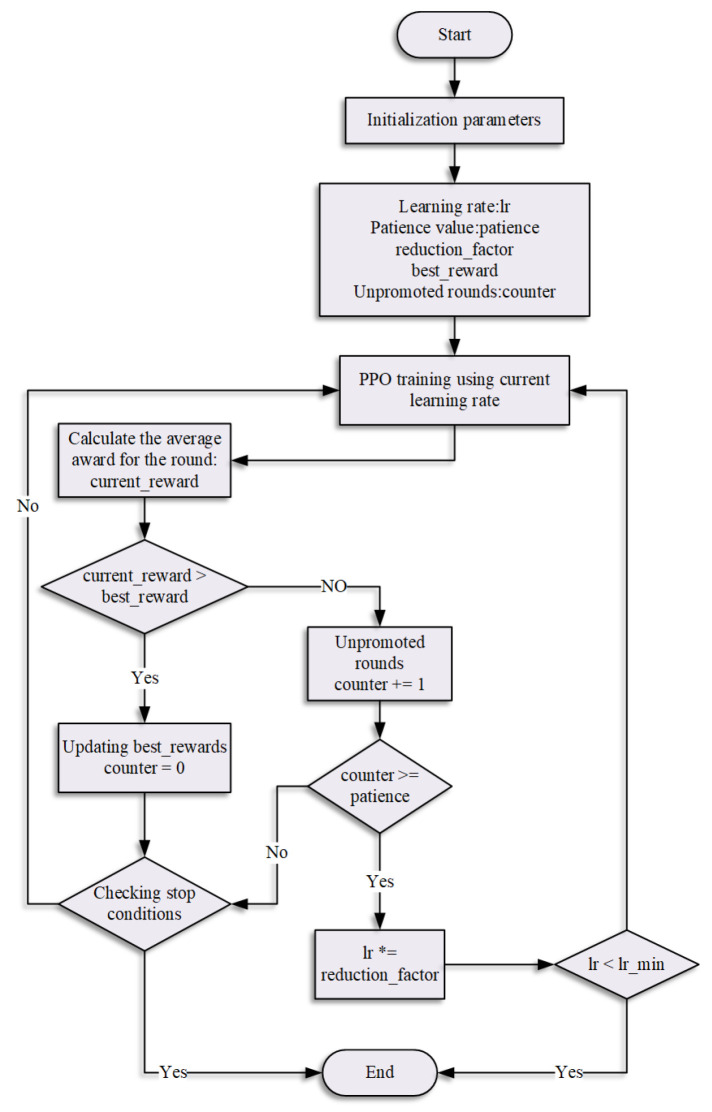
Flow chart of PPO + SA algorithm design.

This design greatly improves the algorithm’s ability to escape from local optima through an initial high learning rate, which enhances the global search capability and effectively prevents convergence to sub-optimal solutions. To achieve stable policy updates, the clipping mechanism in PPO ensures training stability throughout the process. By integrating simulated annealing, the low learning rate at a later stage achieves accurate and stable convergence, achieving an optimal balance between exploration and exploitation. Figure 6 illustrates the detailed workflow of the combined PPO + SA algorithm.

## 5. Experiment of Robotic Arm Trajectory Planning Based on Improved PPO Algorithm

In the simulation experiments, this study employed Stable-Baselines3 [23] and PyBullet [24] as core frameworks. Stable-Baselines3 was utilized to implement both the PPO algorithm and its SA-enhanced variant, while PyBullet served as the physics engine for constructing the three-dimensional simulation environment. These components were integrated through the Gym interface to conduct trajectory planning simulations. Finally the obtained model was applied in a real environment for target grasping.

### 5.1. 3D Simulation Modeling

**Figure 7:**
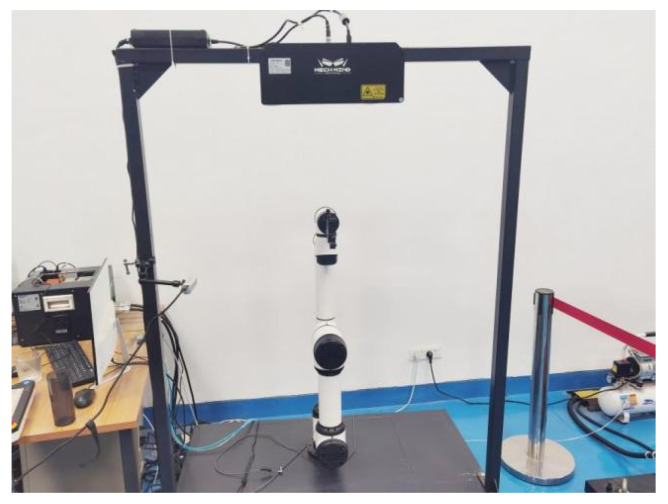
Experimental environment diagram.

A simulation environment is constructed using the Pybullet physics simulation library, which includes an AUBO-I5 robotic arm, a two-finger gripper jaw for the INSPIRE-ROBOTS, and a gantry-fixed depth camera. The robotic arm base is fixed at the origin of the spatial coordinate system. The gantry cross-section is 0.04 m in length and width, and 1.54 m in height, respectively in the left front and right front of the robotic arm, 0.3 m in front, and 0.6 m in the left and right. We build the above simulation environment, 1:1, and then restore the real experimental environment. The real environment is shown in Figure 7:

AUBO-i5 (AUBO Intelligent, Beijing, China) is a nationally certified collaborative robot that complies with the ISO 10218-1 standard. Its 6-Degree-of-Freedom modular joint design achieves a repeatability accuracy of ±0.02 mm and a load capacity of 5 kg, which is crucial for the random object grasping task in this study. Industrial application cases show that it performs well in areas such as loading and unloading materials for machining, welding and assembly, and vision-guided applications. We utilized its native Python control architecture, which was further developed to adapt to the action models generated by the experimental training, thereby meeting the requirements for collision-free random object grasping.

**Figure 8:**
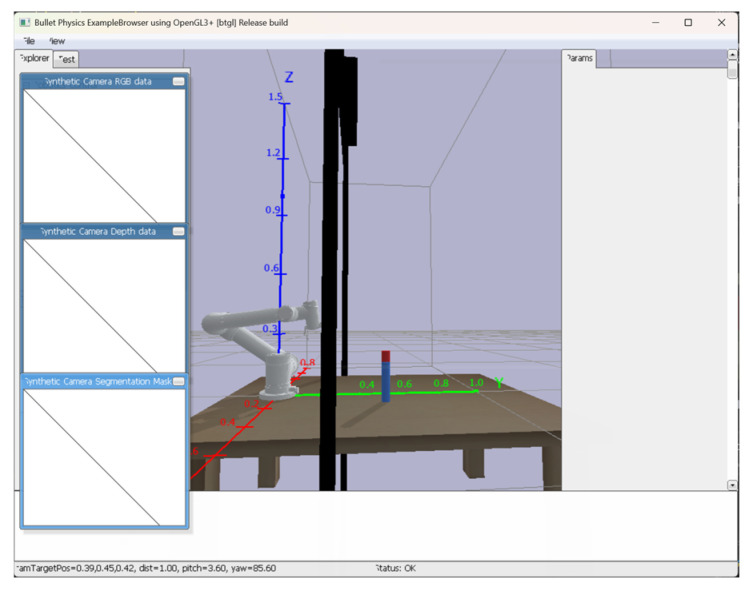
Reinforcement learning simulation training scenario.

Additionally, a rectangular target object (shown in red in the figures) measuring 0.02 m in length and width and 0.05 m in height was placed in the environment for grasping tasks. The cuboid object was selected as the grasping target due to its simple yet stable geometric properties, which make it particularly suitable for evaluating the grasping performance of both the robotic arm and gripper. The complete simulation environment setup is illustrated in Figure 8.

Table 1 describes the hardware and software configurations used in the training.

### 5.2. Grabbing Task Trajectory Planning

**Figure 9:**
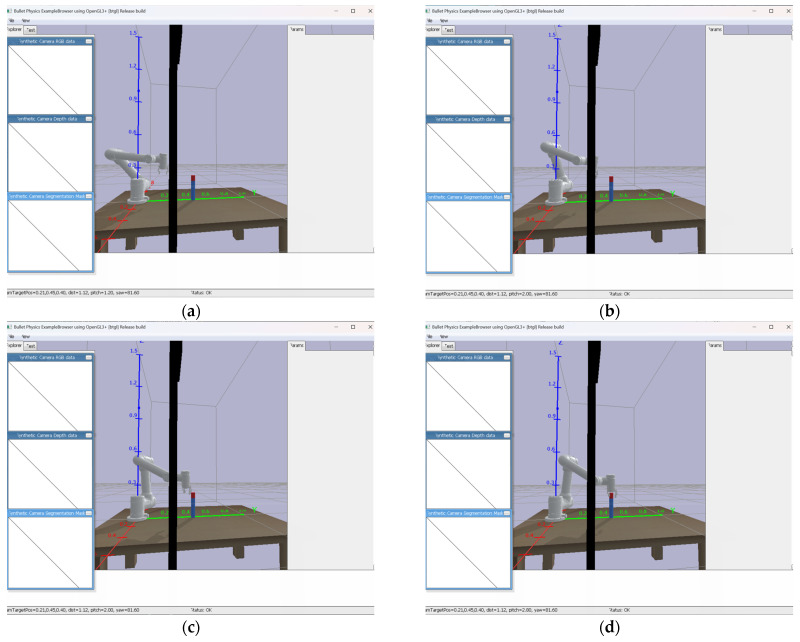
Robotic arm gripping simulation path. (**a**) Initial attitude of the robot arm ready to grasp the target. (**b**) The end of the robot arm leans toward the target position. (**c**) The end of the robot arm is about to reach the target position. (**d**) The end of the robot arm reaches the gripping position.

The path planning of the robotic arm is modeled using DH parameters, imported into the AUBO-i5 kinematic model and converted to a URDF file in order to establish coordinate transformations between joints. The system uses inverse kinematics for the point-to-point motion control of the end-effector, and the arm follows a trajectory from the initial position to the target grasping position. Figure 9a–d illustrate the four key phases of this motion in sequence: initialization, proximity, fine-tuning, and final grasping.

We employ a proximity-aware velocity modulation strategy; when the gripper’s center position enters a threshold near the target, the robotic arm automatically decelerates its motion. This deceleration allows for precise pose adjustment through finer kinematic corrections. This approach slightly increases the number of maneuvering steps, but significantly reduces the risk of collision in cluttered environments compared to a constant velocity.

**Figure 10:**
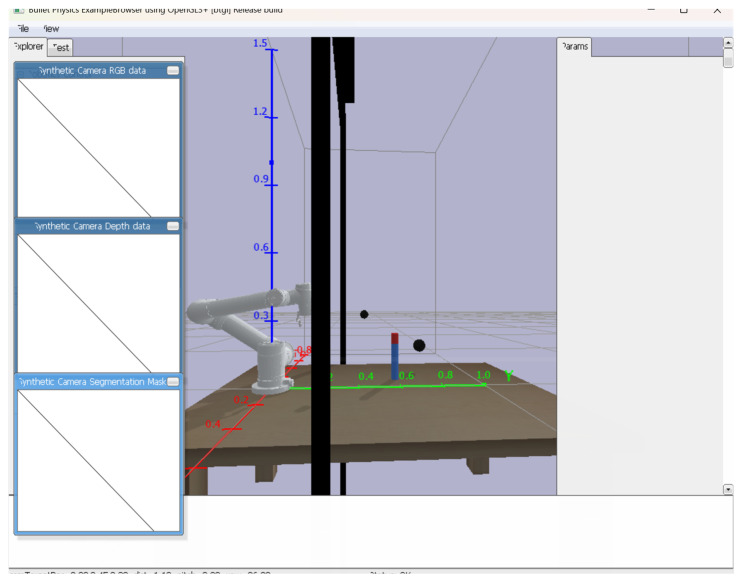
Robotic arm grasping random obstacle generation.

The training program also includes obstacle avoidance adjustment of the robotic arm. During training, two different types of obstacles are encountered: static obstacles (e.g., gantry structures and cameras) that remain stationary throughout the training process, and dynamic obstacles that randomly appear with a probability of 20% during each training session. These dynamic obstacles include primitive shapes such as spheres, cylinders, and cubes, whose sizes and locations are randomized, as illustrated in Figure 10, which shows two spherical obstacles of varying sizes generated in an example scenario. This dual obstacle paradigm enhances the generalization ability of the strategy in terms of collision-free grasping. When randomly generated obstacles are too close to the target object, this training is automatically terminated and these cases are excluded from the policy update by labeling them as “no feasible grasping angle”. For all other cases, the agent learns to bypass the obstacle while completing the grasp. The spatial representations $\left[x_{o},y_{o},z_{o}\right]$ and $\left[\Delta p_{o}\right]$ enable the policy network to encode critical obstacle avoidance information. This geometric awareness is combined with a reward structure that penalizes collisions and proximity states, resulting in collision-free grasping in the test case.

### 5.3. Simulation and Analysis

In this experiment, the process of moving the robotic arm from its initial state to completing the grasping task or reaching the maximum single step length is considered a single grasping process. The advantages of the improved PPO + SA algorithm will be demonstrated by comparing and analyzing the relevant results of the optimized PPO algorithm and the improved PPO + SA algorithm. The specific training parameters are shown in Table 2.

The 20-million-step training was completed within 14.77 h on a single RTX 4070 GPU, achieving 375.8 steps/second throughput with peak GPU utilization of 94.2%. Memory consumption remained at 12.1/16 GB (75.6%), confirming headroom for model complexity scaling. The 2.66 ms/step latency demonstrates feasibility for industrial-scale RL training on cost-effective hardware.

**Figure 11:**
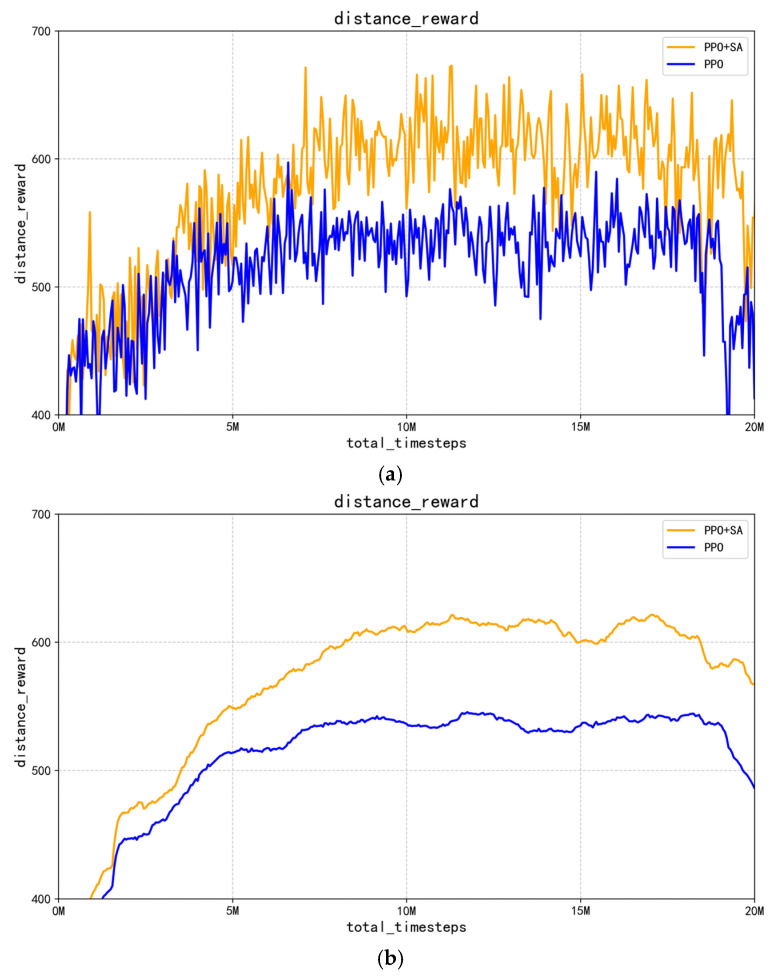
Comparison of robotic arm control process distance rewards. (**a**) Comparison of distance reward curves for PPO and PPO + SA algorithms. (**b**) Comparison of average distance reward curves for PPO and PPO + SA algorithms.

The performance of the optimized PPO algorithm and improved PPO + SA algorithm was evaluated by system simulation experiments. The main indicators included distance reward curves, mean reward curves, grasping success rates, and training efficiency. The distance reward curves and mean reward curves represent the changes in the magnitude of reward values for distance rewards and all rewards (including distance rewards, grasping rewards, and obstacle avoidance rewards) in the training task, respectively. The grasping success rates represent the probability change curve of successful captures in this round of capture actions relative to the total number of capture attempts. The training efficiency represents the change curve in the number of steps required for a single capture action. Figure 11 shows the distance reward curve.

**Figure 12:**
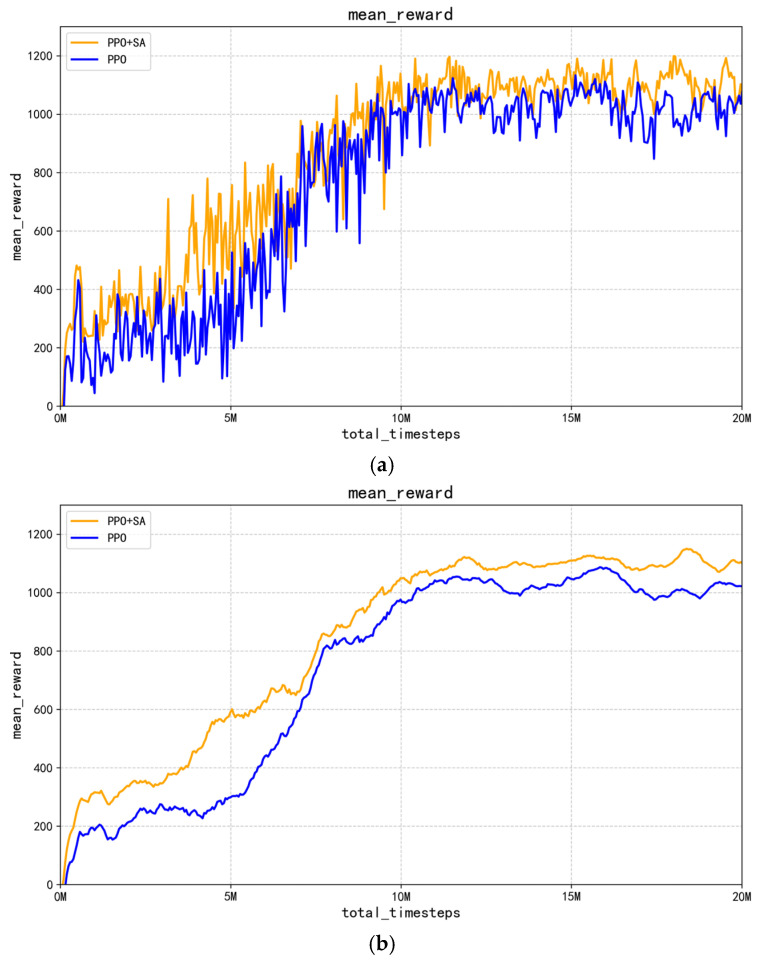
Comparison of robotic arm control process rewards. (**a**) Comparison of PPO and PPO + SA algorithm reward curves. (**b**) Comparison of average reward curves for PPO and PPO + SA algorithms.

In Figure 11a,b, the blue curve represents the optimized PPO algorithm, while the orange curve corresponds to the SA-improved PPO + SA algorithm. As the training progresses, the distance reward value increases steadily and reaches a peak around 13 million interactions, and it then decreases gradually, which is consistent with the dynamic reward parameter scheme defined in our reward function. Figure 11a shows that the improved PPO + SA algorithm exhibits stronger initial oscillations compared to the optimized PPO algorithm, suggesting that it has an enhanced ability to escape from local optima in the early training phase. Furthermore, Figure 11b shows that the improved PPO + SA algorithm achieves higher distance rewards faster by approaching the target more efficiently. Figure 12 shows the corresponding mean reward results.

As shown in Figure 12a,b, the reward value increases gradually as the number of training steps increases. When the number of training steps reaches 10 million, the performance index of the improved PPO + SA algorithm is significantly better than the optimized PPO algorithm. In addition, the average reward accumulation of the improved PPO + SA algorithm shows a steeper upward trend, indicating faster learning progress. Although the improved PPO + SA algorithm gradually stabilizes after 15 million interactions and no longer rises significantly, its reward value is consistently higher than that of the PPO algorithm throughout the training process. This persistent performance gap suggests that improved PPO + SA algorithm has the potential to achieve higher task success rates, providing empirical evidence for its superior effectiveness. The comparative results suggest that the inclusion of simulated annealing in PPO can significantly improve learning efficiency and final policy quality.

**Figure 13:**
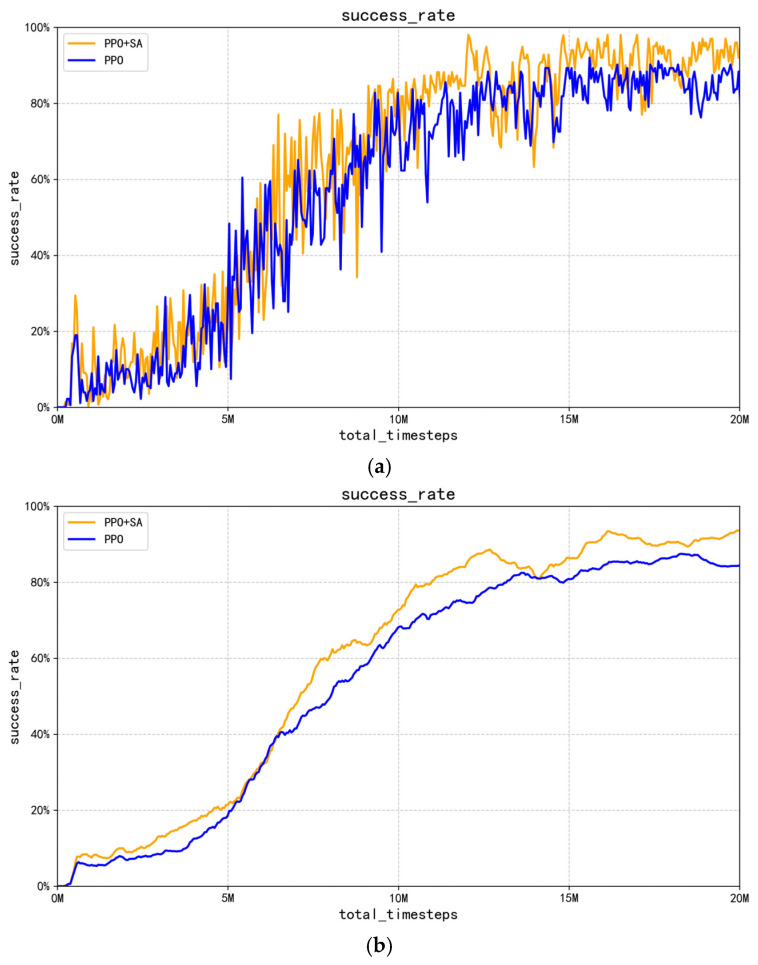
Comparison of success rate of robotic arm gripping processes. (**a**) Comparison of PPO and PPO + SA algorithm crawling success curves. (**b**) Comparison of average crawl success rate between PPO and PPO + SA algorithms.

The reward function values in Figure 12b indirectly indicate the superior success rate of the improved PPO + SA algorithm compared to the optimized PPO algorithm. However, this study provides direct experimental evidence by comparing the final grasping success rates of both algorithms after 20 million training interactions. The conclusive results, presented in Figure 13, demonstrate the following.

Figure 13a,b present comparative success rate curves between the optimized PPO algorithm and improved PPO + SA algorithm throughout 20 million training steps. In Figure 13a, the improved PPO + SA algorithm exhibits significantly larger performance jumps compared to the optimized PPO algorithm during the initial 0–5 million step phase, demonstrating simulated annealing’s effectiveness in escaping local optima. Between 5 and 15 million steps, the improved PPO + SA algorithm maintains a superior exploration capability through its dynamic learning rate adjustment mechanism, enabling faster convergence toward optimal policies. During the final 15–20 million step phase, the algorithm’s simulated annealing component gradually stabilizes the learning rate adjustments, allowing the policy to smoothly converge while maintaining its performance advantage. Figure 13b reveals that the improved PPO + SA algorithm achieves steeper learning progress compared to the optimized PPO algorithm throughout the training process.

**Figure 14:**
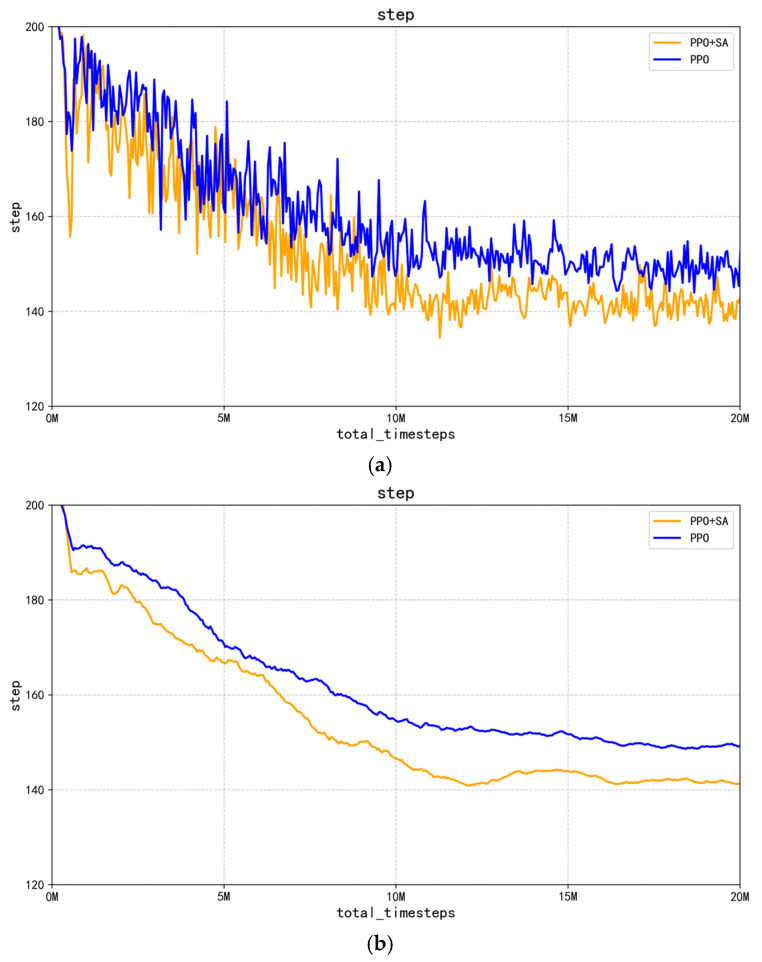
Comparison of single gripping steps of robotic arm. (**a**) Comparison of PPO and PPO + SA algorithms in terms of the number of steps captured in a single run. (**b**) Comparison of average number of steps captured by PPO and PPO + SA algorithms.

This study further evaluates the execution efficiency of the improved PPO + SA algorithm. With the maximum steps per episode set to 200, experimental results demonstrate that the required steps for the robotic arm to reach grasping positions gradually decrease as training progresses. Figure 14 presents a direct comparison between the optimized PPO algorithm and improved PPO + SA algorithm when both have completed 20 million environmental interactions, revealing the following.

As observed in Figure 14b, the comparative fitting curves of the optimized PPO algorithm and improved PPO + SA algorithm after 20 million training steps reveal two key advantages of the enhanced approach. First, the improved PPO + SA algorithm consistently requires fewer steps per episode throughout the entire training process compared to the optimized PPO algorithm. Second, the step-reduction rate of the improved PPO + SA algorithm shows a steeper decline, indicating the faster optimization of movement efficiency.

**Figure 15:**
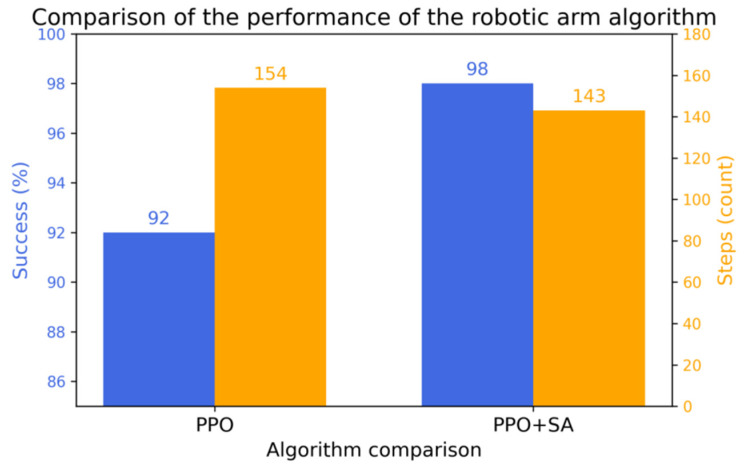
Average success rate vs. average number of steps.

The statistical analysis of the grasping success rates demonstrates that after 20 million steps, the optimized PPO algorithm model achieves a 92% success rate, while the improved PPO + SA algorithm model reaches 98%. Furthermore, the improved PPO + SA algorithm reduces the average steps per successful grasp from the initial 200 steps to 143, outperforming the optimized PPO algorithm’s reduction to 154 steps. These results are visually summarized in Figure 15, which highlights the following.

After the validation of simulation experiments, it can be shown that the improved PPO + SA algorithm is superior to the previous optimized PPO algorithm in terms of distance reward, average reward, success rate of grasping, and the number of steps used to grasp the target. These results confirm that integrating simulated annealing with PPO yields superior convergence properties and accelerated training dynamics. Specifically, the hybrid algorithm achieves the following:

1. Increased ability to jump out of the local optimum at the beginning of training and fast convergence at the later stage;
2. A 6.5% absolute increase in optimal model crawl success rate (98% vs. 92%);
3. A 7.2% reduction in steps per successful episode (143 vs. 154).

### 5.4. Sim-to-Real

The simulation-to-reality (sim-to-real) transfer [25,26] represents a crucial step in deploying robotic reinforcement learning to practical applications. In this study, we enhanced the feasibility of simulation-to-reality transfer through the following measures:

1. Position randomization. We randomly varied target positions and obstacles during training to improve the model’s generalization ability. This randomization helps the model adapt to complex scenarios in the real world caused by different target positions;
2. Adaptive noise injection. We implemented adaptive noise injection in the observation space and introduced noise into joint angle measurements to simulate the uncertainty of sensors in the real world;
3. Standardization of the unified observation space. We achieved consistent standardization between simulated and real-world observations to avoid inference failures caused by inconsistent observation spaces;
4. Coordinate System Transformation. Accurate coordinate transformation is achieved through hand-eye calibration to ensure consistency between the real and simulated worlds.

**Figure 16:**
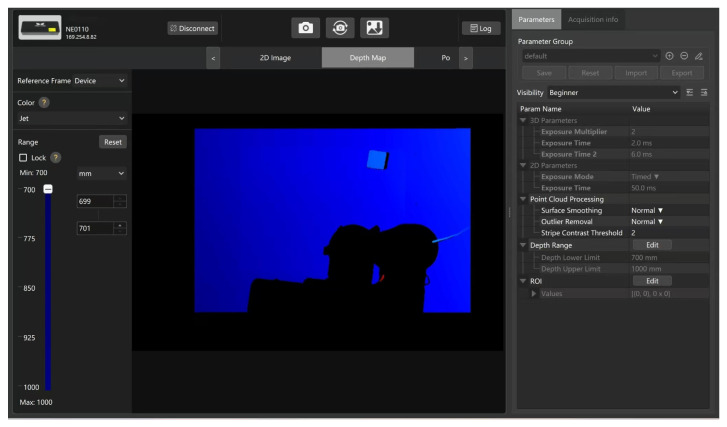
Mech-Eye depth map.

For target object data acquisition, this experiment utilizes the Mech-Mind depth camera (Mech-Eye Pro M Enhanced), which captures 2D images, 3D data, and point clouds, while the point cloud provides coordinates relative to the camera frame, as shown in Figure 16.

The training process uses the robotic arm’s base coordinate system. To maintain consistency, hand–eye calibration is performed to obtain the coordinate transformation between the camera and the robotic arm base. The hand–eye calibration result in this study is represented by a 4 × 4 homogeneous transformation matrix $T_{\mathrm{cam}}^{\mathrm{base}}$, as shown in Equation (13).

(13) $$T_{\mathrm{cam}}^{\mathrm{base}}=\left[\begin{array}{cccc} -0.9961 & 0.0219 & 0.0854 & -162.08\\ 0.0241 & 0.9994 & 0.0246 & -403.37\\ -0.0848 & 0.0265 & -0.9960 & 1408.24\\ 0 & 0 & 0 & 1 \end{array}\right]$$

The matrix $T_{\mathrm{cam}}^{\mathrm{base}}$ is used to transform point $p_{\mathrm{cam}}$ from the camera coordinate system to the robotic arm’s base coordinate system as $p_{\mathrm{base}}$, with the transformation formula given by Equation (14).

(14) $$p_{\mathrm{base}\;}=R\;\times p_{\mathrm{cam}}+t$$

In this formulation, R (top-left 3 × 3 submatrix) represents the rotation matrix, while t (top-right 3 × 1 vector, units: mm) denotes the translation vector, with the last row serving as homogeneous coordinate padding. Through this transformation, any point’s coordinates in the camera frame can be converted to the robotic arm’s base frame for model inference. Obstacle information is similarly acquired using the identical transformation.

**Figure 17:**
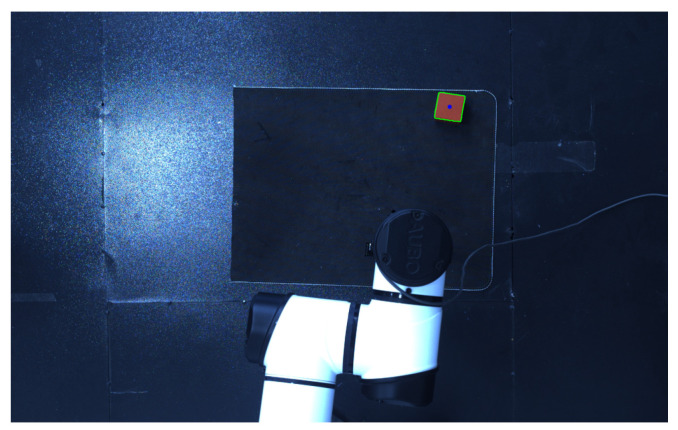
YOLO Calibration of the center point of the target object.

During the process of robotic arm grasping, the detection of the target is accomplished by YOLO, which can mark the pixel position of the center point of the object to be grasped. Then, based on the point cloud, it is converted into coordinates. The actual marking effect is shown in Figure 17.

The robotic arm’s state information is acquired through built-in servo mechanisms at each joint. The AUBO-i5 robotic arm features integrated encoders within every joint’s servo motor, enabling the real-time measurement of motor shaft rotation angles. Based on forward kinematics modeling, the controller converts these joint angles into the end-effector’s spatial pose (position and orientation), forming the essential data foundation for physical grasping operations.

**Figure 18:**
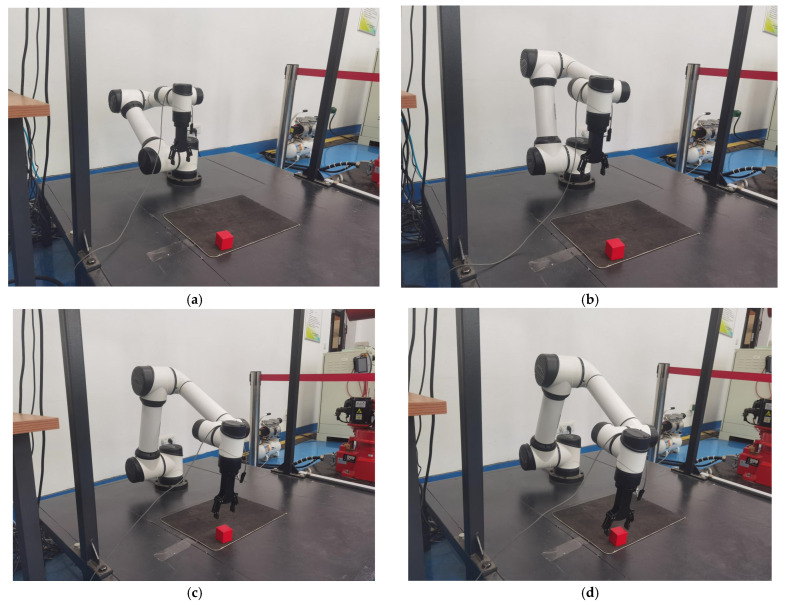
Diagram of the grasping process in a real environment through model reasoning. (**a**) The robotic arm is in the initial position. (**b**) The robotic arm is approaching the target position. (**c**) The robotic arm is about to reach the target position. (**d**) The robotic arm reaches the grasping position.

The crawling process in the real environment is shown in Figure 18.

It is demonstrated in Figure 18, in that the real environment, it is possible to perform actions acquired by reasoning to accomplish a random grasping function similar to the simulation.

**Figure 19:**
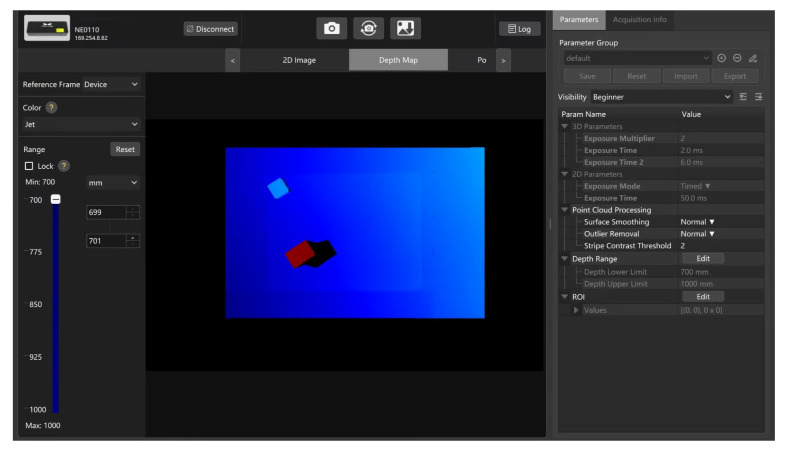
Mech-Eye depth map with obstacles.

We also tested placing obstacles in the environment, which undoubtedly made grasping the target much more difficult. Similar to grasping a random target without obstacles, the Mech-Mind depth camera acquired the information and coordinates of the target and obstacles and converted them into the robot arm’s reference coordinates. Figure 19 shows the depth map obtained after adding obstacles, with blue representing the target to be grasped and red representing obstacles.

**Figure 20:**
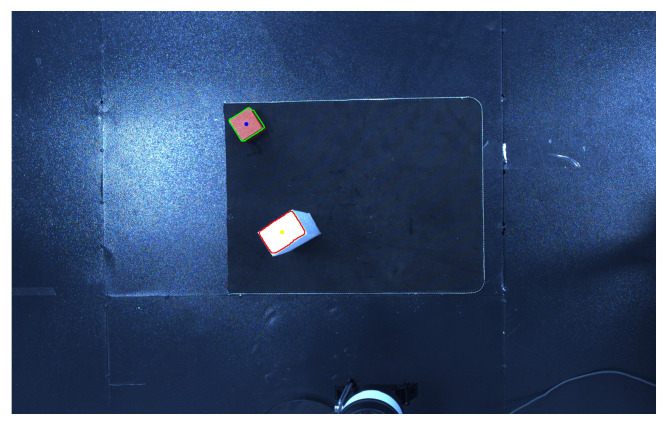
YOLO calibration map containing obstacles.

Figure 20 is a YOLO calibration image containing obstacles. In this image, the white objects outlined in red are obstacles, while the red objects outlined in green are the targets to be captured. From this image, the coordinates of the targets and obstacles can be obtained, and the distance from the robot arm joints to the obstacles can be calculated using these coordinates. This is one of the parameters required in the state space and represents very important data for obstacle avoidance and grasping operations.

**Figure 21:**
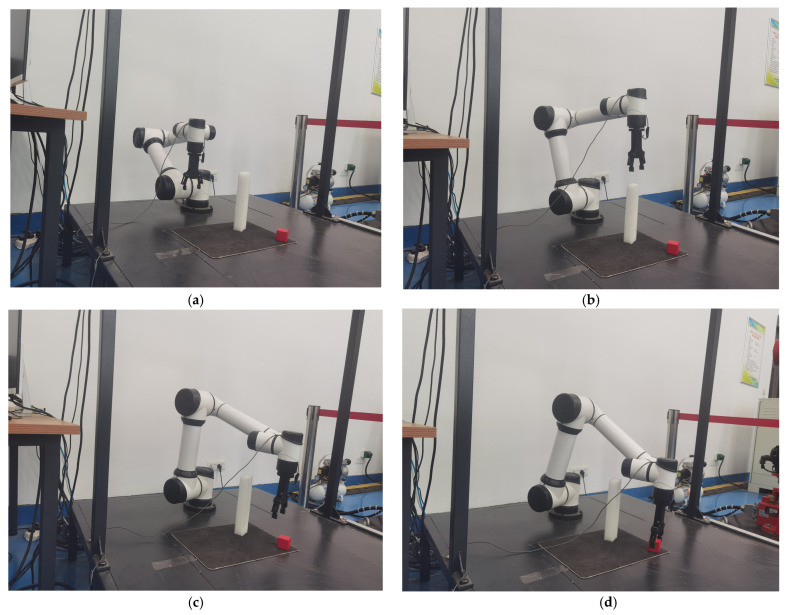
Schematic diagram of obstacle avoidance and grasping in a real environment through model inference. (**a**) The robotic arm is in the initial position. (**b**) The robotic arm is avoiding obstacles and approaching the target position. (**c**) The robotic arm has successfully avoided obstacles and is about to reach the target position. (**d**) The robotic arm reaches the grasping position.

Similar to obstacle-free grasping, with this data, model inference and robotic arm operation can be performed to achieve obstacle avoidance grasping consistent with the simulated environment. The obstacle avoidance grasping process is shown in Figure 21.

Video S1 demonstrates the robot arm performing target grasping and obstacle avoidance grasping operations in a real-world environment.

### 5.5. Future Work

Although the current PPO algorithm integrated with the simulated annealing algorithm has achieved encouraging results, further optimization can be achieved by introducing other reinforcement learning methods. For example, the curriculum learning [27] framework adopts an incremental training paradigm to systematically increase task difficulty. In this experiment, curriculum learning can be applied to gradually expand the generation range of target objects. This integration is expected to bring dual benefits, as follow: (1) Accelerated convergence– by deferring complex obstacle avoidance to a later stage, the algorithm focuses early training cycles on basic grasping skills, which is estimated to reduce training time in the initial phase. A simplified initial task allows for the faster stabilization of the strategy before complexity is introduced. (2) Enhancing universality—the staged difficulty increment builds robust feature representations, improves model generalization, and enhances learning for complex tasks.

The algorithm has achieved good results in grasping actions, but in order to more clearly demonstrate its applicability and robustness in various operating environments, future research will involve testing the proposed algorithm on a variety of different tasks (such as placement, assembly, or collaborative tasks).

## 6. Conclusions

This study focuses on the PPO algorithm for reinforcement learning in continuous action spaces. The research begins with a detailed introduction to the PPO algorithm, followed by the design of simulation strategies encompassing the robotic arm’s state representation, action space configuration, loss function formulation, and reward structure. Subsequently, the simulated annealing (SA) algorithm is presented along with a feasibility analysis of its integration with PPO. Through comparative experiments between the optimized PPO algorithm and improved PPO + SA algorithms, key performance metrics including average grasping reward, success rate, and step efficiency are systematically evaluated. The simulation results demonstrate that incorporating simulated annealing for dynamic learning rate adjustment yields comprehensive performance improvements. Furthermore, the implemented model successfully transitions from simulation to physical deployment, achieving the precise grasping of randomly positioned targets in real-world scenarios. The experimental outcomes validate the effectiveness of the proposed approach in both virtual and physical environments while maintaining operational robustness.

Subsequent experiments will continue to introduce concepts from other reinforcement learning algorithms and adjust the parameter relationships between PPO, SA, and other reinforcement learning algorithms. This is expected to accelerate model convergence speed and enhance its generalization capabilities. The experiments will be continuously optimized and upgraded, tested across various tasks to adapt to more complex and diverse scenarios, meet the future needs of smart factories, and improve current production efficiency levels.

## Figures and Tables

**Table 1:** Software and hardware configuration for training.

Software and Hardware	Detailed Information
Processors	Intel I7-12800HX
Discrete Graphics Card	NVIDIA GeForce RTX 4070
Operating technique	Windows-11
Physical Simulation Library	Pybullet
Deep Reinforcement Learning Library	Stable-Baselines3: 2.0.0
Python Environment	3.10.16
Customizing the Environment to Create Libraries	Gymnasium: 0.28.1

**Table 2:** Hyper-parameters of the PPO + SA algorithm.

Parameter	Value
max_step (Maximum number of execution steps)	20,000,000
initial_lr (Initialized learning rate)	0.0003
min_lr (Minimum learning rate)	0.000001
annealing_coefficient	0.98
batch_size (Number of samples in a single training session)	256
clip_range=	0.2
Gmma (discount factor)	0.99
n_steps (Number of environmental steps collected per update)	2048
n_epoches (Number of training rounds executed per update)	10
Single_length_max (Maximum length of a single training session)	200

## Data Availability

The original contributions presented in this study are included in the article/Supplementary Materials. Further inquiries can be directed to the corresponding author.

## Supplementary Materials

The following supporting information can be downloaded at: https://www.mdpi.com/article/10.3390/s25175253/s1, Video S1: Demonstration of robotic arm target grasping and obstacle avoidance grasping in a real environment.
